# Disrupted Emergent Properties of the Brain in Schizophrenia: Insight From Integrated Information Decomposition of Resting State fMRI

**DOI:** 10.1002/brb3.71352

**Published:** 2026-03-31

**Authors:** Livio Tarchi, Lorenzo Lasagni, Leonardo Ubaldi, Jessica Bottacin, Enrico Lodovici, Annalisa Di Giacomo, Luca Zompa, Tiziana Pisano, Andrea Bianchi, Ludovico D'Incerti, Giovanni Castellini, Valdo Ricca

**Affiliations:** ^1^ Department of Neuroscience, Psychology, Drug Research and Child Health University of Florence Florence Italy; ^2^ Psychiatry Unit, Department of Health Sciences University of Florence Florence Italy; ^3^ Neuroimaging Unit, Department of Neuroscience and Human Genetics Meyer Children's Hospital IRCCS Florence Italy; ^4^ Children and Adolescence Psychiatry, Department of Neuroscience and Human Genetics Meyer Children's Hospital IRCCS Florence Italy

**Keywords:** computational psychiatry, integrated information theory, psychosis, resting state fMRI

## Abstract

Background: Schizophrenia is a severe psychiatric disorder marked by specific cognitive and clinical disturbances, for which neuroimaging biomarkers remain elusive. Novel theoretical and computational frameworks, such as integrated information decomposition, offer promising approaches to provide interpretable biomarkers for neuroimaging alterations in schizophrenia, potentially capturing disruptions relevant to consciousness and self‐experience.

Methods: In this preliminary methodological exploration study, resting‐state functional MRI (rsFMRI) data from 72 patients with schizophrenia and 74 healthy controls were retrieved and analyzed. Integrated information decomposition was leveraged to assess pairwise brain connectivity according to redundant, transferred, and synergistic components of information processing, as well as an overall metric of emergent consciousness/information integration: Φ. Clinical correlates with the Positive and Negative Syndrome Scale and the Wechsler Adult Intelligence Scale were assessed by partial Spearman correlations. Diagnostic accuracy was assessed through L1‐regularized logistic regressions, after 5‐fold cross‐validation.

Results: Redundancy was positively correlated with intelligence quotient (IQ) across both groups (rho = 0.187, *p*‐value = 0.033). Within patients, information metrics were positively correlated with stereotyped thinking (min rho = 0.343, max *p*‐value = 0.006) and preoccupation (min rho = 0.250, max *p*‐value = 0.046). Positive symptoms were positively correlated with redundancy (min rho = 0.250, max *p*‐value = 0.047). Promising diagnostic accuracy was reached with Φ (balanced accuracy = 64.38%, area under the curve = 70.89%), redundancy (balanced accuracy = 84.93%, area under the curve = 92.30%), and synergy (balanced accuracy = 65.75%, area under the curve = 70.93%).

Conclusions: These preliminary findings suggest that information metrics may offer clinically relevant, interpretable biomarkers for schizophrenia.

## Graphical abstract

Integrated Information Decomposition reveals altered patterns of brain information processing in schizophrenia, with disrupted synergy, redundancy, transfer, and integration (Φ) linked to cognitive and clinical features. These metrics show potential as interpretable neuroimaging biomarkers for diagnostic classification and understanding the emergent properties of consciousness in psychiatric disorders.

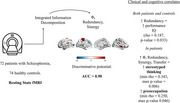

## 1 Introduction

Schizophrenia is a complex mental disorder, characterized by cognitive, affective, and behavioral impairments, including delusions, hallucinations, and other perceptual disturbances (American Psychiatric Association 2022). While schizophrenia only affects around 0.3% of the general population at the global level (Charlson et al. 2018), this disorder significantly contributes to a substantial burden of disease across most countries around the world (Same et al. 2024).

Several factors contribute to this significant burden. First, while pharmacological treatments are effective (Tarchi et al. 2024), and while timely treatment may improve long‐term outcomes (Penttilä et al. 2014), substantial diagnostic delays still persist to the present day (Chen et al. 2020), impacting the successful remission and recovery for a large number of patients. Second, while biological alterations have long been described for schizophrenia (Howes and Kapur 2009), clinical practice still lacks valid diagnostic and/or therapeutic biomarkers.

Historically, neuroimaging studies have attempted to identify reliable biomarkers for schizophrenia, mainly focusing on alterations in the dopaminergic system as either diagnostic standards (Kapur 2003) or indicators of treatment response (Nordström et al. 1993). However, the current scientific consensus posits that a mono‐aminergic perspective of the disorder insufficiently captures the complexity of schizophrenia (Stahl 2018). In this perspective, resting‐state MRI studies offer the possibility to investigate not only specific brain regions (possibly associated with neurochemical signaling, i.e., dopaminergic), but also whole‐brain dynamics which may underlie the disorder (Damiani et al. 2024, 2022).

In fact, if schizophrenia is conceptualized as a disorder at least partly characterized by perceptual disturbances (Kapur 2003), integrated frameworks are necessary to better grasp the potential fragmentation within and between neural centers dedicated to cognitive, affective, or behavioral control (Yokosawa 2014). In brief, integrated frameworks posit that complex psychological phenomena, such as consciousness, perception, and lived experiences overall, may be better described as *emergent* properties of specific biological substrates (e.g. the central nervous system) (Pessoa 2022). In other words, integrated frameworks suggest that such phenomena (and their disturbances) cannot be fully understood by examining individual components alone (i.e., neurons or ensemble of neurons), but rather through their dynamic and complex interaction (Pessoa 2022).

In light of these considerations, studies capable of describing how specific biomarkers relate to clinical features are timely needed, in order to bridge the gap between clinical and preclinical studies. For this purpose, neuroimaging studies may need to employ dimensions of integrated information processing (Tononi et al. 2016), rather than focusing on the functional connectivity between brain areas, or between a specific area and the rest of the brain (Pessoa 2022). This endeavor may be facilitated by recent developments in the formal description of information processing in distributed systems, specifically for time series, namely integrated information decomposition (Luppi et al. 2021; Mediano et al. 2021; Varley 2023).

**FIGURE 1:**
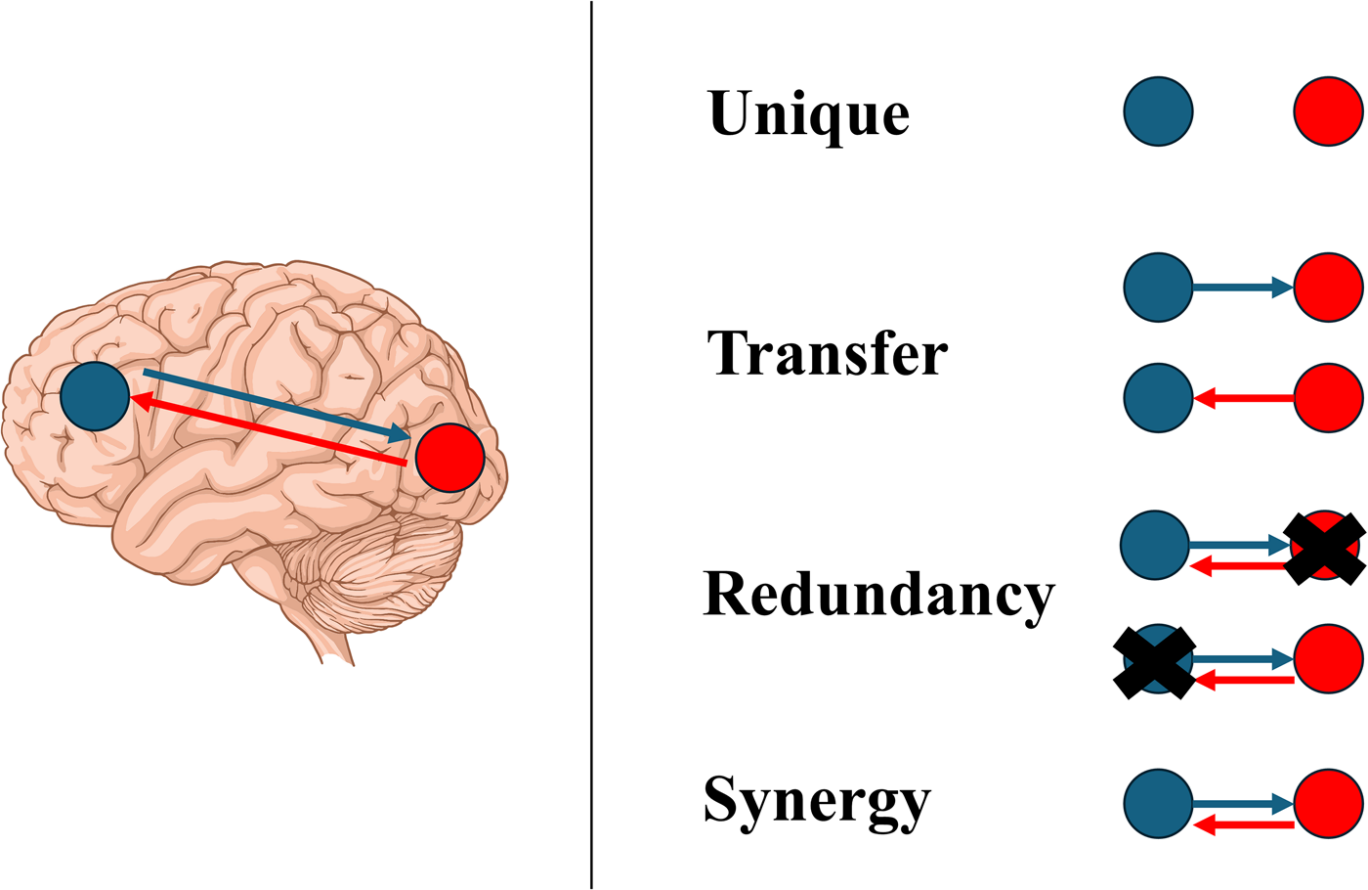
Consciousness and integrated information decomposition. A graphical representation of specific measures derived from integrated information decomposition. Once unique, transfer, redundancy, and synergy are defined, Φ, a measure of integrated consciousness, can be computed (synergy + transfer − redundancy). Lateral view of the brain, illustration from NIAID NIH BioArt Source (bioart.niaid.nih.gov/bioart/60; NIAID Visual & Medical Arts, 2024).

In neuroimaging studies, the framework of integrated information decomposition allows a better description of how two brain regions interact, beyond classic measures such as functional connectivity (Luppi et al. 2024). Indeed, integrated information decomposition measure how two brain regions interact with each other through time, according to different sources of information, namely the unique contribution of each region in the dyadic interaction, the redundant information provided by both regions, the information transferred from one region to the other (and vice‐versa), as well as the amount of synergistic integration—in other words, information available only when the two regions interact (Luppi, Mediano, et al. 2024). See Figure 1 for a graphical representation of information metrics as derived by integrated information decomposition.

Notably, once transfer, redundant, and synergistic interactions are computed, an overall measure of integrated information processing and consciousness can be described (Luppi et al. 2021):

Φ=Synergy+Transfer−Redundancy

However, while similar frameworks have since been adopted to better understand consciousness and its disturbances, through empirical derivations of Φ, including from resting state fMRI data (Luppi et al. 2022; Luppi, Mediano, et al. 2024), to the authors’ knowledge, similar approaches have yet not been attempted for disturbances of complex psychological phenomena, such as psychiatric disorders, beyond what was previously attempted by computational models alone (Silverstein et al. 2017).

### 1.1 Aims

Based on these premises, the present preliminary methodological exploration study aims to characterize, for the first time, how integrated information (Φ) is altered in individuals with schizophrenia, positing lower integrated information (Φ) within patients in comparison to controls. Moreover, the present study aims to investigate clinical correlates of integrated information and consciousness thus derived (Φ), as well as the diagnostic potential of integrated information (Φ), to evaluate its potential as a biomarker for schizophrenia.

## 2 Materials and Methods

### 2.1 Sample and Participants

The sample was collected from the Center for Biomedical Research Excellence (COBRE), and was composed of 72 patients with schizophrenia and 74 healthy controls (ranging from 18 to 65 years old). Participants had been excluded from the study if they had any neurological disorders, severe head trauma with more than 5 min loss of consciousness, history of substance abuse or dependence within the last 12 months. Diagnostic information for this database was collected using the Structured Clinical Interview for DSM Disorders (SCID). Further information can be found in the parent study (Mayer et al. 2013) and at https://fcon_1000.projects.nitrc.org/indi/retro/cobre.html.

### 2.2 Image Acquisition

All images were collected on a 3 Tesla Siemens Trio scanner. Functional MRI data were collected using a T2*‐weighted echo‐planar imaging (EPI) sequence with the following parameters: TR = 2 s, TE = 29 ms, number of slices = 32, flip angle = 75°, voxel size = 3 × 3 × 4 mm, and matrix 64 × 64. An anatomical scan was acquired for each participant, in order to align the functional data to the anatomical brain. The resting state fMRI scan lasted approximately 6 min. More information on image acquisition can be found at https://fcon_1000.projects.nitrc.org/indi/retro/cobre.html.

### 2.3 Preprocessing

Preprocessing of resting‐state data was performed in accordance with the previous literature on integrated information decomposition applications for resting state fMRI data (Luppi, Mediano, et al. 2024). The functional imaging data were preprocessed using a standard pipeline, implemented within MATLAB version 2023b and SPM12‐based (http://www.fil.ion.ucl.ac.uk/spm) toolbox CONN (http://www.nitrc.org/projects/conn), version 22.u2407. The pipeline includes standard pipelines, aimed for reproducibility in fMRI research (Botvinik‐Nezer et al. 2020).

First, five scans are removed to account for magnetization artifacts before reaching the steady state. Second, images are realigned, and motion is estimated and corrected. Then, slice‐timing correction is performed to account for differences in time of acquisition between slices. Outlier timepoints for the presence of motion are flagged for subsequent regression. Anatomical and fMRI scans are co‐registered. Images are spatially normalized to the Montreal Neurological Institute (MNI‐152) standard space with 2 mm isotropic resampling resolution, using segmented grey matter masks, and a priori grey matter templates.

Physiological and motion artifacts were denoised with CompCor, as implemented by CONN. Linear detrending and band‐pass filtering (0.008 to 0.09 Hz) were applied. As global signal regression (GSR) may introduce anti‐correlation artifacts (Power et al. 2014), in line with previous literature (Damiani et al. 2020; Luppi, Mediano, et al. 2024), no GSR was applied.

### 2.4 Integrated Information Decomposition

In accordance with previous literature on integrated information decomposition applications for resting state fMRI data (Luppi, Mediano, et al. 2024), brains were parcellated into 232 cortical and subcortical regions of interest (ROIs), obtained from the Schaefer atlas (Schaefer et al. 2018), augmented by cerebellar ROIs (Tian et al. 2020). The timecourses of denoised BOLD signals were averaged between all voxels belonging to a given atlas‐derived ROI, using the CONN toolbox, and then extracted. Standard SPM methods were used to deconvolve the hemodynamic response function from the regional BOLD signal timeseries prior to analysis.

Integrated information decomposition of timeseries was then through specific software previously released (Mediano et al. 2021; ΦID—Integrated Information Decomposition, 2025). To see how to derive unique, transfer, redundant, and synergistic measures, as well as Φ from single information decomposition “atoms”, see Luppi et al. 2021. Three matrices were computed. The first was obtained by first retrieving each pairwise information metric, then averaging by diagnostic group (group‐average matrices). The second, similarly to previous works, averaged each pairwise information metric per region, for each participant (individual region‐wise matrices; (Luppi, Mediano, et al. 2024). The third further averaged all brain regions, to derive one information metric per participant (global information matrices).

### 2.5 Informed Consent and Ethical Approval

All procedures contributing to this work complied with the ethical standards of the relevant national and institutional committees on human experimentation and with the Helsinki Declaration of 1975. All procedures involving human subjects/patients were approved by local institutional bodies (Mayer et al. 2013).

### 2.6 Statistical Methods

Descriptive statistics were first computed by means and standard deviations. Group differences were estimated by the Mann‐Whitney test for continuous variables, and Fisher exact test for categorical variables. Group differences in global information metrics were computed by analysis of covariance (ANCOVA), adjusting for potential confounding factors (age, sex, handedness, intelligence quotient—IQ). Prior to conducting ANCOVA, the following assumptions were tested: (i) normality of residuals; (ii) homogeneity of variance was confirmed via Levene's test (F‐value = 0.513, *p*‐value = 0.475); (iii) linearity of the relationship between the covariate and dependent variable was examined through scatterplot inspection; and (iv) homogeneity of regression slopes was verified by testing the interaction term between the covariate and group. Among both patients and controls, clinical correlates were investigated with verbal, performance, and full IQ derived from the Wechsler Adult Intelligence Scale (WAIS) (Wechsler 2012), after adjustment for age, sex, and handedness. Among patients only, clinical correlates were also investigated by partial Spearman's rho coefficients, after adjustment for age, sex, handedness, and full IQ, with items from the Positive and Negative Syndrome Scale (PANSS) (Kay et al. 1987). Items from PANSS were then interpreted considering the original loading onto the original three domains (Kay et al. 1987), namely: positive, negative, and general psychopathological symptoms. Logistic regression was computed after L1 regularization, after 5 fold cross‐validation, as implemented by scikit‐learn (Pedregosa et al. 2011). L1 regularization was adopted in order to perform feature selection, as L1 regularization favors sparse solutions for regression coefficients.

Analyses were performed using R 4.3.3 (R Core Team 2024), R Studio 2025.05.0 + 496 (RStudio Team 2024), Python 3.10.11 (Python Software Foundation 2025), JASP 0.19.3 (JASP Team 2025).

## 3 Results

**FIGURE 2:**
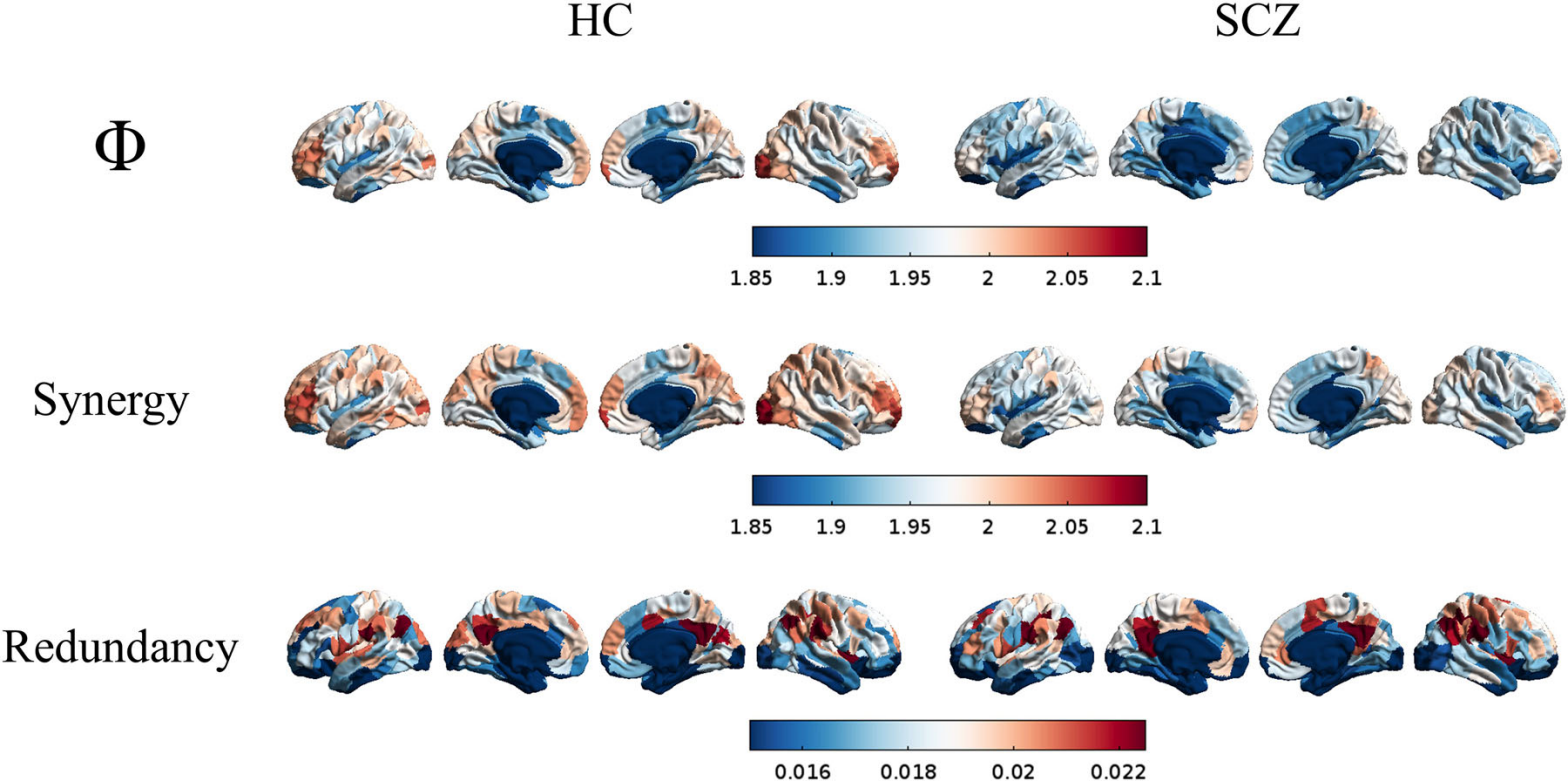
Information metrics, for each region, by diagnostic group. Subcortical and cerebellar regions were included in the analyses, but were here not shown. Red: higher information. Blue: less information. Information metrics by arbitrary units. HC = Healthy controls, SCZ = patients with schizophrenia.

The final sample was composed of 146 individuals, mostly balanced between patients with schizophrenia and healthy controls. Participants were mostly male, and healthy controls reported higher verbal, performance, and full IQ in comparison to participants with schizophrenia. Further descriptive statistics were reported in Table 1. No significant difference was observed for global information metrics. No significant difference was observed also when controlling for age, sex, handedness, or IQ (Supplementary Table S1). See Figure 2 for a graphical representation of information metrics by diagnostic groups across brain regions.

**TABLE 1:** Sample descriptives.

	Healthy controls (n = 74)	Patients with schizophrenia (n = 72)	Mann‐Whitney U test (*p*‐value)	Fisher's exact test (*p*‐value)
Age	35.71 ± 11.62	38.17 ± 13.89	0.380	/
Sex	females: 23 males: 51	females: 14 males: 58	/	0.129
WASI verbal IQ	106.41 ± 11.01	97.87 ± 16.73	<0.001	/
WASI performance IQ	113.74 ± 12.19	102.67 ± 16.64	<0.001	/
WASI full scale IQ	111.33 ± 11.61	99.59 ± 16.86	<0.001	/
Age at first psychotic symptoms	/	21.03 ± 7.42	/	/
Age at first psychiatric hospitalization	/	22.32 ± 7.73	/	/
Number of psychiatric hospitalizations	/	5.39 ± 5.38	/	/

*Note*: ± Standard deviations. *Legend*: PANSS = Positive and Negative Symptoms Scale; WASI = Wechsler Abbreviated Scale of Intelligence.

### 3.1 Clinical Correlates

In both patients and controls, the global redundancy of information between regions was positively correlated with performance IQ (rho = 0.187, *p*‐value = 0.033). This relationship was not significantly different between patients and controls (interaction factor, linear regression model, group by WASI; *p*‐value = 0.897).

In patients, positive symptoms were positively correlated with redundancy (delusions: rho = 0.250, *p*‐value = 0.047; conceptual disorganization: rho = 0.286, *p*‐value = 0.022; grandiosity: rho = 0.262, *p*‐value = 0.037; suspiciousness: rho = 0.303, *p*‐value = 0.015; Supplementary Materials Figure S1). Among negative symptoms, all information metrics were positively correlated with stereotyped thinking (Φ: rho = 0.343, *p*‐value = 0.006; redundancy: rho = 0.359, *p*‐value = 0.004; synergy: rho = 0.347, *p*‐value = 0.005; transfer: rho = 0.347, *p*‐value = 0.005; Supplementary Materials Figure S2). For general psychopathology, all information metrics were positively correlated with preoccupation (Φ: rho = 0.283, *p*‐value = 0.023; redundancy: rho = 0.250, *p*‐value = 0.046; synergy: rho = 0.287, *p*‐value = 0.021; transfer: rho = 0.279, *p*‐value = 0.026). Moreover, Φ and synergy positively correlated with somatic concern (rho = 0.318, *p*‐value = 0.011 and rho = 0.323, *p*‐value = 0.009, respectively), while redundancy with guilt feelings (rho = 0.276, *p*‐value = 0.027), “lack of judgement and insight” (rho = 0.291, *p*‐value = 0.020), and active social avoidance (rho = 0.305, *p*‐value = 0.014; Supplementary Materials Figure S3). No information metric was significantly associated with other clinical factors, such as age at first symptoms, age at first admission, or number of hospitalizations.

### 3.2 Discriminative Potential

First, global information metrics revealed only moderate discriminative potential (Table 2). To be noted, L1 regularization favored a model discarding transfer information (regression coefficients, Φ = −10.41, redundancy = 20.62, synergy = 7.34).

**TABLE 2:** Discriminative potential.

	Global information metrics	Φ	Redundancy	Transfer	Synergy
Balanced accuracy	56.16%	64.38%	84.93%	50.00%	65.75%
Area under the curve	60.34%	70.89%	92.30%	50.00%	70.93%

*Note*: Logistic regression, L1 regularized, cross‐validated by 5 folds.

**FIGURE 3:**
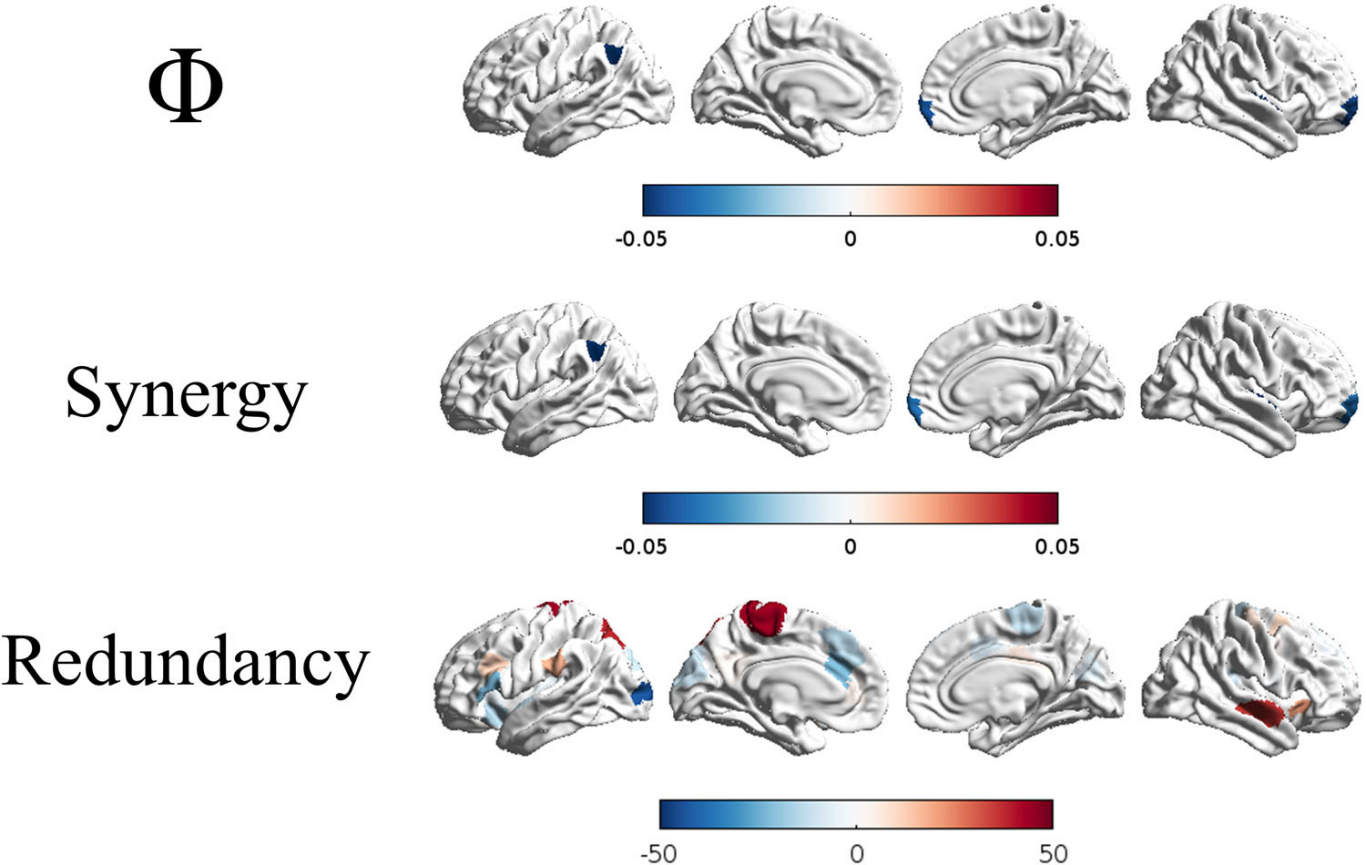
Brain regions contributing to diagnostic accuracy to discriminate between patients with schizophrenia and healthy controls. Subcortical and cerebellar regions were included in the analyses, but were here not shown. Red: schizophrenia more likely. Blue: control more likely. The strength of association was measured by logistic regression coefficients. To be noted, L1 regularization achieves sparse solutions, with a minimal set of brain regions significantly contributing to logistic regression.

By contrast, logistic regression, as computed on individual metrics, across parcellated brain regions, reached substantial discriminative potential for Φ (5 fold cross‐validated results, three significantly predictive regions, including the bilateral prefrontal cortex and the right Wernicke's area; Supplementary materials Table S2), redundancy (co‐localized in the same regions as Φ; Supplementary Materials Table S2), and synergy information (5 fold cross‐validated results, thirty significantly predictive regions, including bilateral striatal regions, primary and supplementary somato‐motor areas, as well as the left prefrontal cortex, the left anterior cingulate cortex, the right medial and posterior paracingulate cortex, the right insula; Supplementary Materials Table S2). Transfer did not significantly predict diagnostic categories (Table 2). See Figure 3 for a graphical representation of brain regions contributing to diagnostic predictions.

## 4 Discussion

This study represents the first application of a comprehensive set of information metrics, derived from integrated information decomposition, to schizophrenia, aiming to characterize altered brain dynamics, as related to consciousness, in patients with a psychiatric disorder. While no significant group differences emerged at the global level, region‐specific alterations were evident and showed meaningful associations with cognitive and clinical dimensions. Moreover, region‐specific alterations were shown to accurately classify participants as either patients with schizophrenia or healthy controls, mostly by leveraging lower integrated information (Φ), lower synergy, and disrupted, albeit mostly higher, redundancy in patients with schizophrenia.

In fact, reduced redundancy in the prefrontal cortex, paracingulate gyri, and primary somato‐motor or visual areas was associated with higher odds of being classified as a patient with schizophrenia. To be noted, one previous work, already described reduced synergy in patients with schizophrenia in comparison to controls (Ding et al. 2024). However, no insight over redundancy or Φ was described (Ding et al. 2024). In current results, higher redundancy in secondary somato‐motor areas and the insula was associated with higher odds of being classified as a patient with schizophrenia. These results suggest potential diverging contributions for regions within different networks, as well as from redundancy in primary sensory (higher in controls) in contrast to associative regions (higher in patients with schizophrenia).

Current results highlight how global perspectives of information dynamics may not yield clinically relevant information for patients with schizophrenia. Rather, schizophrenia may involve specific disruptions in the distribution and organization of information processing, consistent with previous literature emphasizing regional dysfunctions in prefrontal and temporoparietal areas (Gong et al. 2020; Merola et al. 2024, Merola et al. 2026). Notably, however, global redundancy information was positively associated with IQ scores across both groups. This association aligns with recent models emphasizing the role of distributed computation and network integration in favoring fluid intelligence, including verbal and performance tasks (Duncan, Assem, and Shashidhara 2020).

The positive correlation between global redundancy and cognitive performance suggests that redundant information, potentially within specific brain regions, may support cognitive performance. Redundancy within the frontoparietal cortex and regions of the executive control network may favor information robustness—as multiple regions sharing similar information could facilitate compensation, error correction, and consistent cognitive output. Importantly, the lack of significant interaction with group status indicates this relationship may be transdiagnostic or dimensionally preserved across psychiatric and non‐psychiatric populations.

However, redundancy also emerged as a core discriminant between patients with schizophrenia and controls, with higher values in specific brain regions indicating a greater probability of being diagnosed with schizophrenia. Taken together, these findings suggest that proper allocation of neural resources may facilitate cognitive performance, whereas a shift of redundancy from primary sensory to associative regions may favor cognitive disruptions. Similar findings were recently reported for state‐dependent reconfigurations in resting state brain dynamics for schizophrenia, as measured by global signal correlation coefficients (Damiani et al. 2024). This previous work showed that inefficient and disrupted allocations of neural resources to specific brain regions were correlated with lower performance at cognitive tasks in patients with schizophrenia (Damiani et al. 2024). However, to the authors’ knowledge, current results are the first report of correlations between information metrics derived from integrated information decomposition and performance IQ.

To the authors’ knowledge, present results show for the first time a significant correlation between metrics derived from integrated information decomposition and clinical psychiatric features. In fact, within patients with schizophrenia, stereotyped thinking, a core feature of negative symptoms in psychosis (Maurage et al. 2017; Ricarte, Del Rey, Ros, Latorre, and Berna 2018), was positively associated with all information metrics. These associations suggest that patients with more severe disorganization symptoms may exhibit inefficient or noisy information integration, which aligns with prior work implicating fronto‐temporal dysconnectivity in thought disorders (Stripeikyte et al. 2021) or psychosis (Maderthaner et al. 2023). Beyond stereotyped thinking, a pattern for a similar trend of association with clinical features was also observed for preoccupation, suggesting that disruptions in information integration may not only be associated with formal thought disruptions (stereotyped or restrictive cognition), but also with distressing lived experiences, at least in patients with schizophrenia. Moreover, Φ and synergy were positively correlated with general psychopathology symptoms (i.e., somatic concern). By contrast, higher redundancy was associated with lower clinical insight, maladaptive social isolation and negative affect (i.e., PANSS: “lack of judgment and insight”, social avoidance, and guilt feelings). This result further suggests that higher redundancy within brain regions usually not dedicated to cognitive performance tasks may favor inefficient error compensation and inconsistent cognitive output in patients with schizophrenia. This diverging pattern of association between information metrics also indicates that distinct clinical characteristics may be associated with each of these components of information integration (on one hand: Φ and synergy, on the other: redundancy).

Despite the lack of appreciable diagnostic accuracy from global information metrics alone, region‐wise information metrics revealed promising discriminative capacity. Logistic regression identified key contributions from prefrontal and temporoparietal regions, particularly for Φ and synergy information. These metrics—representing integrative computations only possible through joint regional activity—were also distributed across distant cortical areas, including regions that have previously been implicated in salience processing and the default mode network (Schaefer et al. 2018), thus being potentially implicated in aberrant salience (Aloi et al. 2024; Corlett and Fraser 2025) and lack of coherent self‐related experiences in schizophrenia (Raballo et al. 2021). The differential diagnostic performance observed across information metrics provides indirect evidence against the interpretation that the reported clinical correlations are primarily artifactual, albeit preliminary in nature. Specifically, if a global confound—such as non‐specific variance in the BOLD signal, possibly due to motion artifacts—were driving the associations, global metrics or regional transfer may have yielded comparable diagnostic accuracy. Instead, the markedly superior performance of redundancy relative to transfer, which showed no diagnostic utility, suggests that the metrics are capturing genuinely distinct and neurobiologically meaningful dimensions of brain connectivity, also given the specific regional distribution of driving regions for classification algorithms.

While future studies are certainly needed before a wider generalization of current results, present findings align with and extend a growing body of literature employing information‐theoretic and entropy‐based approaches to characterize resting‐state fMRI alterations in schizophrenia (Blair et al. 2024; Maksymchuk et al. 2025; Niu et al. 2023; Parente 2026; Xue et al. 2019; Yang et al. 2015). Notably, studies employing multiscale entropy analyses have consistently documented reduced entropy and reduced integration in schizophrenia (Blair et al. 2024; Parente 2026), which can be interpreted as reflecting a loss of adaptive neural flexibility—a construct conceptually adjacent to redundancy observed in the present sample. As partly previously described, the specific association between stereotyped thinking and all four information metrics is particularly noteworthy in light of prior work on reduced entropy in the default mode network (Maksymchuk et al. 2025; Xue et al. 2019), altered entropy in higher‐order interaction within the cognitive‐control domain (Maksymchuk et al. 2025), as well as prior work on reduced entropy and cognitive alterations in patients with schizophrenia (Blair et al. 2024). Collectively, these convergences suggest that integrated information decomposition captures dimensions of neural information processing that are both statistically and neurobiologically meaningful, potentially sensitive to the clinical heterogeneity characteristic of patients with schizophrenia.

In summary, current results provide empirical support for integrated frameworks in psychiatry, while also suggesting that disturbances in emergent psychological phenomena ‐ such as consciousness or coherent self‐experience ‐may arise from altered interactions among distributed brain regions (Fornito et al. 2017; Pessoa 2022; Segal et al. 2023; Tononi et al. 2016). The regional specificity of Φ, synergy, and redundancy disruptions in schizophrenia offers a potential mechanistic account for fragmented consciousness, consistent with phenomenological accounts of psychosis as dis‐integrative in nature (Ebisch and Aleman 2016; Stanghellini et al. 2016, Stanghellini et al. 2020). In this perspective, a recent work seems to confirm that integrated information decomposition may help better describe spatiotemporal dynamics of brain connectivity in patients with schizophrenia or bipolar disorder (Li et al. 2025). Indeed, Li and colleagues showed profound disruptions in brain dynamics of these patients within the psychosis spectrum, with evidence of widespread alterations in the balance between redundant and synergistic information (Li et al. 2025). While adopting a different methodological framework (e.g., multiscale connectivity networks, analyses of higher‐order interaction, different clinical population, as well as only focusing on redundancy and synergy), these results lend credible support to the hypothesis that information metrics may finally provide a bridge between neuroimaging and phenomenology by offering interpretable and clinically relevant diagnostic biomarkers.

### 4.1 Limitations

While the sample size was balanced and derived from a well‐characterized dataset, future studies may employ larger populations, aiming to reach higher heterogeneity in sociodemographic characteristics of enrolled participants, including better representation of non‐male participants. Current results were cross‐sectional in nature, and future longitudinal studies may explore whether information metrics derived from integrated information decomposition may yield stable biomarkers for the diagnosis, prognosis, or prediction of treatment‐response in schizophrenia. Future studies may also employ task‐based MRI studies to better assess the potential of information metrics, such as Φ, synergy, and redundancy, to capture alertness, vigilance, and salience detection. Clinical correlates were not adjusted for multiple comparisons. Reported results should then cautiously be interpreted as explorative and preliminary in nature, mainly aimed to be hypothesis‐generating for future replication in larger and independent samples.

Future studies may also better explore whether higher redundancy may be characterized as either adaptive or maladaptive according to overall brain dynamics, specific tasks, as well as specific brain regions. In fact, patients with schizophrenia were here more accurately discriminated by redundancy, rather than an overall measure of integration such as Φ, suggesting at least partial compensation for redundant information within overall brain dynamics, and thus a potential for higher redundancy to be conceptualized as adaptive in nature.

## 5 Conclusions

In this preliminary methodological exploration study, integrated information decomposition showed promising potential to be an interpretable biomarker in patients with schizophrenia. These findings support the utility of integrated information frameworks in psychiatry and underscore the importance of adopting multivariate, system‐level approaches to understanding complex psychiatric phenomena.

## Author Contributions

**Livio Tarchi**: conceptualization; methodology; formal analysis; investigation; data curation; writing – original draft, writing – review and editing; visualization. **Lorenzo Lasagni**: conceptualization; methodology; formal analysis; investigation; data curation; writing – original draft. **Leonardo Ubaldi**: conceptualization; methodology; validation; investigation; data curation; writing – original draft. **Jessica Bottacin,Enrico Lodovici, Annalisa Di Giacomo, and Luca Zompa**: conceptualization; investigation; data curation; writing – review and editing. **Tiziana Pisano, Andrea Bianchi, Ludovico D'Incerti, Giovanni Castellini, and Valdo Ricca**: conceptualization; resources; writing – review and editing; supervision; project administration.

## Funding

This work was supported by #NEXTGENERATIONEU (NGEU) and funded by the Ministry of University and Research (MUR), National Recovery and Resilience Plan (NRRP), project MNESYS (PE0000006)—a multiscale integrated approach to the study of the nervous system in health and disease (DN. 1553—DN. 11.10.2022). Funding included expenses related to personnel. No influence on research results was exerted by the funding agency. Open access funding provided by Università degli Studi di Firenze within the Wiley ‐ CRUI ‐ CARE Agreement.

## Ethics Statement

All procedures contributing to this work complied with the ethical standards of the relevant national and institutional committees on human experimentation and with the Helsinki Declaration of 1975. All procedures involving human subjects/patients were approved by local institutional bodies (Mayer et al. 2013)

## Supporting information




**Supplementary Material**: brb371352‐sup‐0001‐SuppMat.docx

## Data Availability

Further information on COBRE can be found in the parent study (Mayer et al. 2013) and at https://fcon_1000.projects.nitrc.org/indi/retro/cobre.html. The datasets generated during the current study are available from the corresponding author on reasonable request.
