# Supplementary material for: Disrupted Emergent Properties of the Brain in Schizophrenia: Insight From Integrated Information Decomposition of Resting State fMRI
Source: Brain Behav. 2026 Mar 31;16(4):e71352. doi: 10.1002/brb3.71352 (PMC13111988; doi:10.1002/brb3.71352)
Supplement: Supplementary file 1 — Supplementary Material: brb371352‐sup‐0001‐SuppMat.docx [file BRB3-16-e71352-s001.docx]

**Supplementary Materials**

| **Table S1 - Group differences, patients with schizophrenia compared to healthy controls** | | | | | | |
| --- | --- | --- | --- | --- | --- | --- |
|  | **Age adjusted** | **Age and handedness adjusted** | **Age and sex adjusted** | **Age and verbal IQ adjusted** | **Age and performance IQ adjusted** | **Age and full IQ adjusted** |
| Φ | 3.049  (p-value 0.083) | 3.505  (p-value 0.063) | 3.366  (p-value 0.069) | 0.820  (p-value 0.367) | 0.909  (p-value 0.342) | 0.345  (p-value 0.558) |
| Redundancy | 0.105  (p-value 0.746) | 0.274  (p-value 0.601) | 0.001  (p-value 0.981) | 0.219  (p-value 0.640) | 0.633  (p-value 0.428) | 0.887  (p-value 0.348) |
| Synergy | 2.939  (p-value 0.089) | 3.360  (p-value 0.069) | 3.284  (p-value 0.072) | 0.775  (p-value 0.380) | 0.844  (p-value 0.360) | 0.307  (p-value 0.581) |
| Transfer | 0.001  (p-value 0.999) | 0.001  (p-value 0.999) | 0.001  (p-value 0.999) | 0.001  (p-value 0.999) | 0.001  (p-value 0.999) | 0.001  (p-value 0.999) |
| *Note*: in each cell, F-value and p-value for analysis of covariance. | | | | | | |

**Figure S1 – Clinical correlates, patients only. Positive symptoms scale from PANSS.**In each cell is reported the partial spearman rho coefficients, adjusted for age, sex, handedness and full IQ. The figure is color coded (darker blue = stronger positive coefficient; darker red = stronger negative coefficient).
Legend: *** p < 0.001, ** p < 0.01, * p < 0.05.


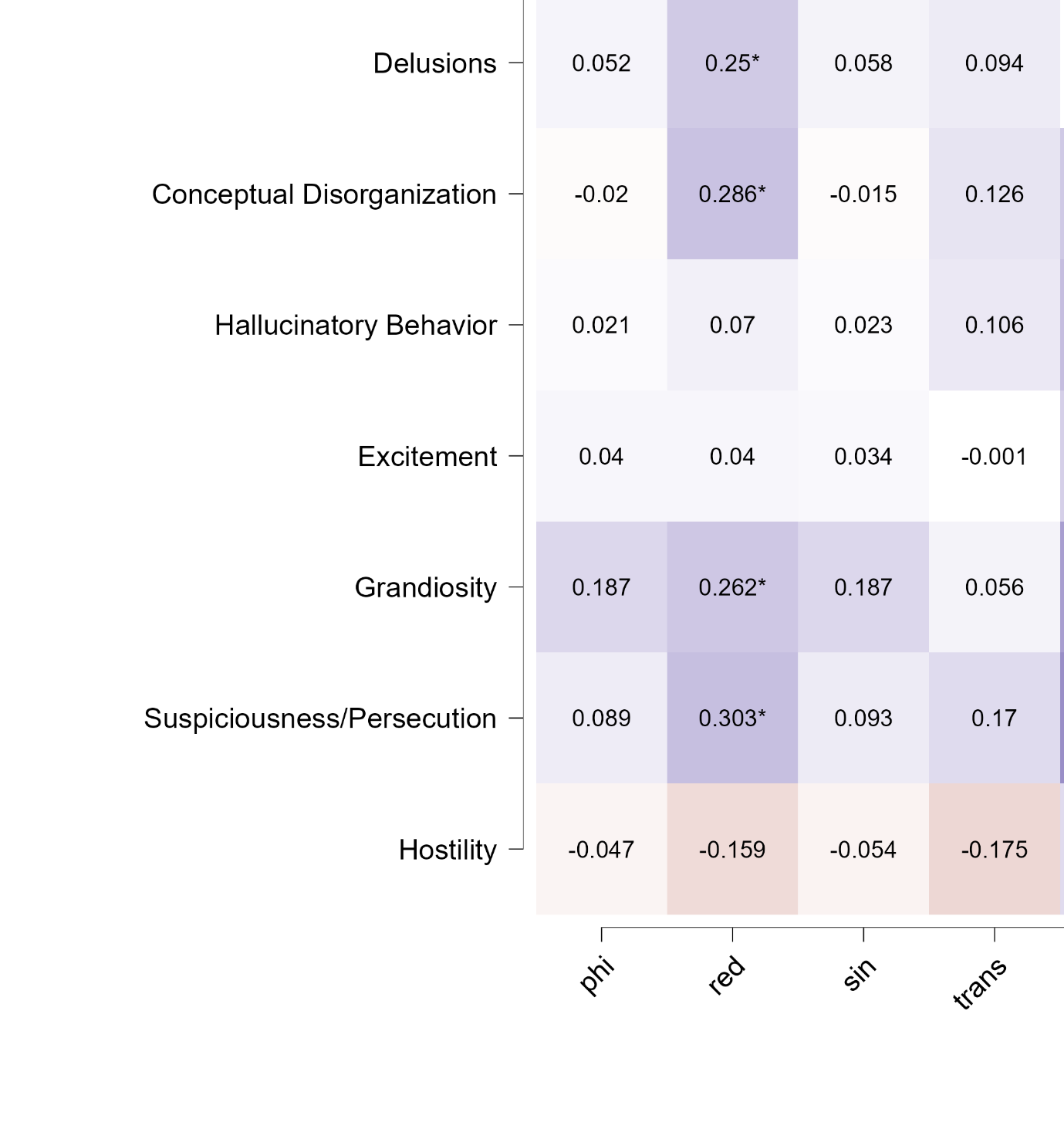


**Figure S2 – Clinical correlates, patients only. Negative symptoms scale from PANSS.**In each cell is reported the partial spearman rho coefficients, adjusted for age, sex, handedness and full IQ. The figure is color coded (darker blue = stronger positive coefficient; darker red = stronger negative coefficient).
Legend: *** p < 0.001, ** p < 0.01, * p < 0.05.

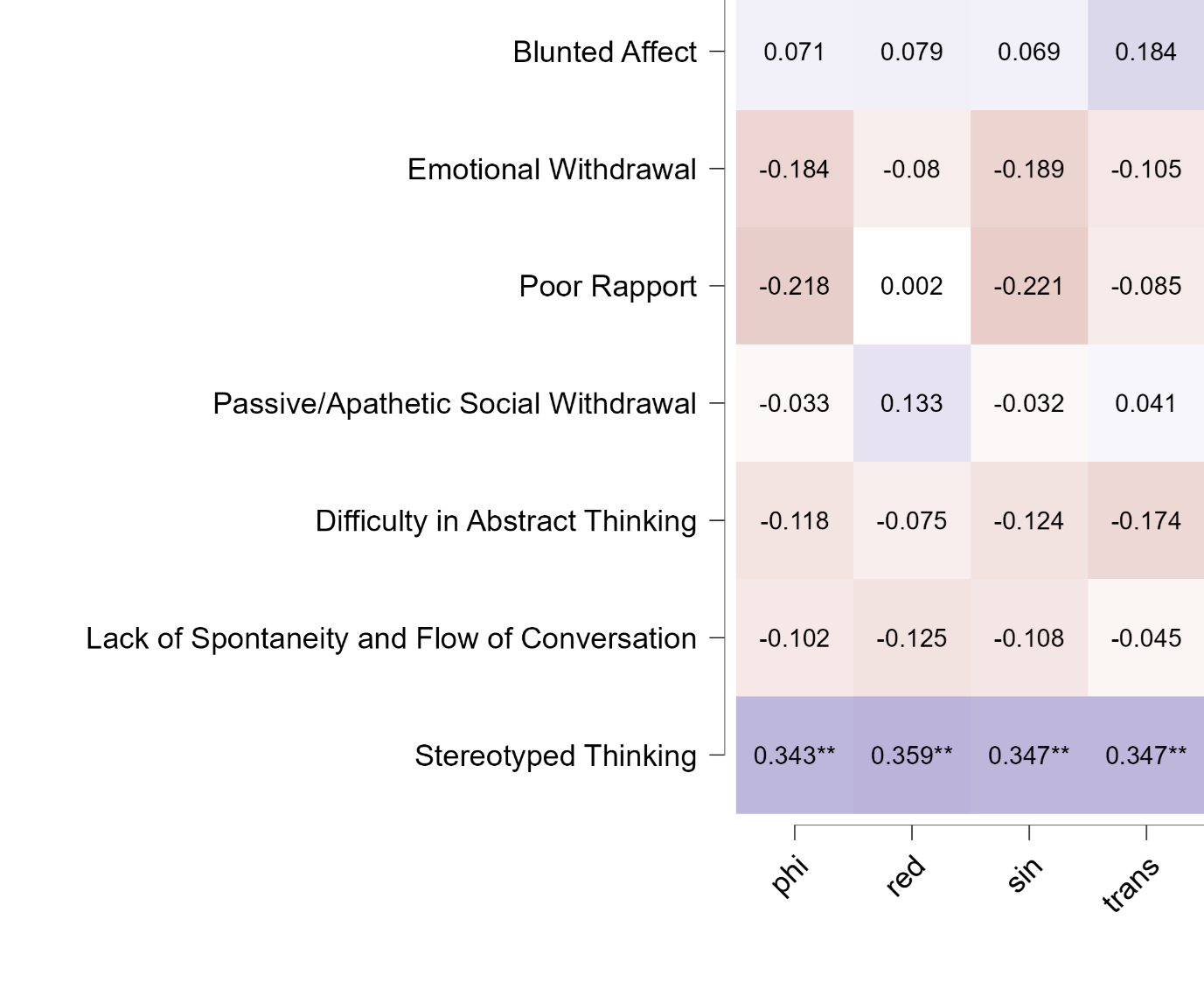


**Figure S3 – Clinical correlates, patients only. General psychopathology from PANSS.**In each cell is reported the partial spearman rho coefficients, adjusted for age, sex, handedness and full IQ. The figure is color coded (darker blue = stronger positive coefficient; darker red = stronger negative coefficient).
Legend: *** p < 0.001, ** p < 0.01, * p < 0.05.

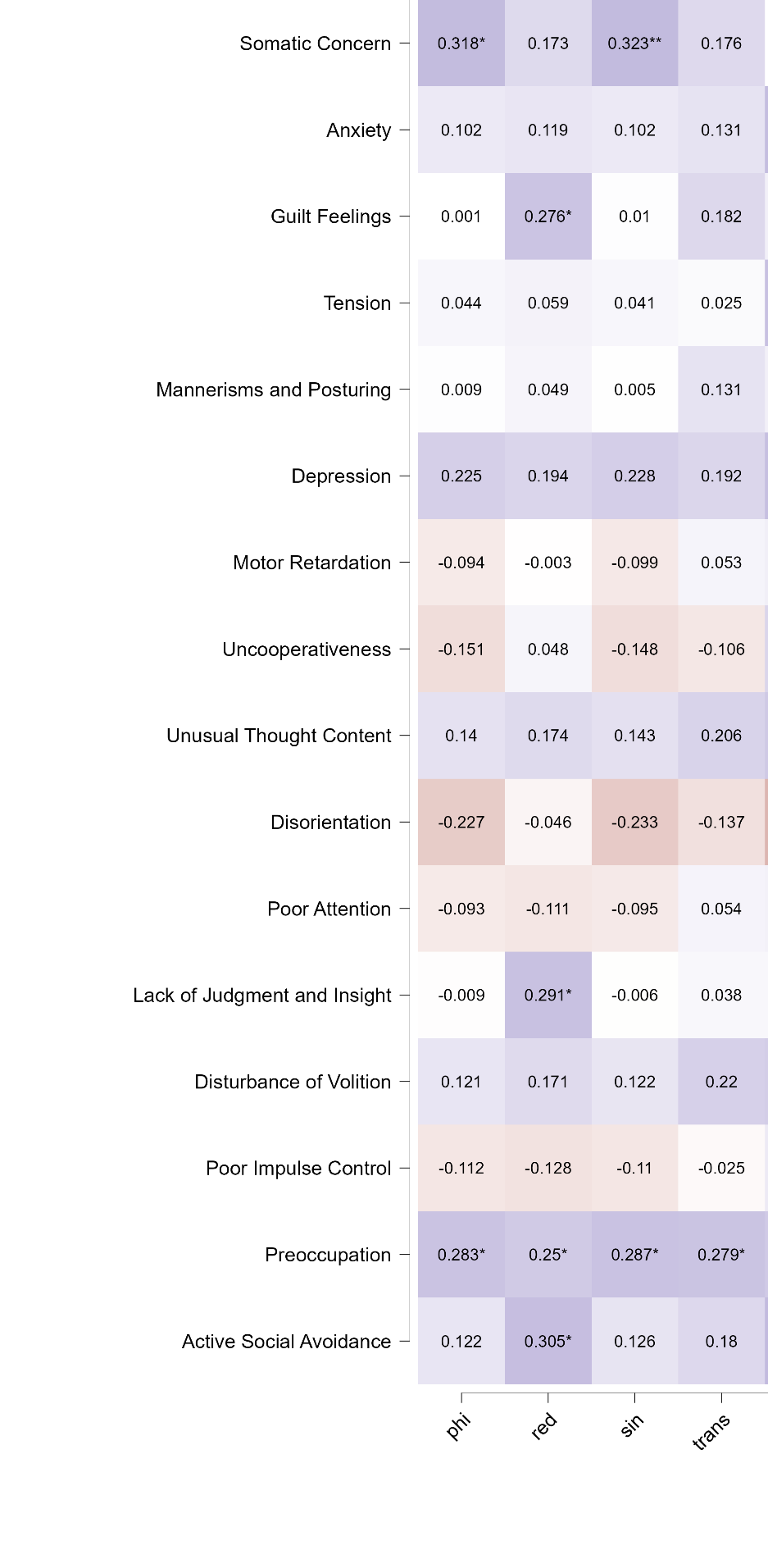


| **Table S2 - Regions discriminating between patients with schizophrenia and healthy controls** | | | |
| --- | --- | --- | --- |
|  | Φ | **Redundancy** | **Synergy** |
| Left dorsal prefrontal cortex 1 | -0.264 | / | -0.250 |
| Right lateral prefrontal cortex 2 | -0.416 | / | -0.455 |
| Right temporal pole 1 | -0.044 | / | -0.037 |
| Left extra-striate superior | / | -42.890 | / |
| Left somato-motor area 1 | / | -11.350 | / |
| Left somato-motor area 3 | / | -12.914 | / |
| Left somato-motor area 5 | / | -4.097 | / |
| Left temporal occipital 1 | / | 43.054 | / |
| Left post central 3 | / | 37.525 | / |
| Left frontal medial 1 | / | 17.625 | / |
| Left lateral prefrontal cortex 1 | / | -3.355 | / |
| Left temporal pole 3 | / | 5.053 | / |
| Left lateral prefrontal cortex 1 | / | 11.880 | / |
| Left temporal 1 | / | -14.623 | / |
| Left inferior parietal lobule 1 | / | -22.298 | / |
| Left dorsal prefrontal cortex 1 | / | 3.892 | / |
| Left dorsal prefrontal cortex 3 | / | -21.989 | / |
| Left ventral prefrontal cortex 1 | / | -13.845 | / |
| Left retrosplenial 1 | / | 2.927 | / |
| Right extra-striate superior 6 | / | -0.289 | / |
| Right somato-motor area 1 | / | -7.399 | / |
| Rightsomato-motor area 7 | / | -10.899 | / |
| Right somato-motor area 8 | / | -1.696 | / |
| Right somato-motor area11 | / | -2.146 | / |
| Right supplementary somato-motor area | / | 7.351 | / |
| Right superior parietal lobule 1 | / | -12.688 | / |
| Right insula 3 | / | 9.817 | / |
| Right inferior parietal lobule 1 | / | 2.382 | / |
| Right medial posterior prefrontal cortex 1 | / | -7.898 | / |
| Right lateral prefrontal cortex 2 | / | 20.660 | / |
| Right lateral ventral prefrontal cortex 2 | / | 6.187 | / |
| Right medial prefrontal cortex 1 | / | 35.300 | / |
| Right retrosplenial 1 | / | -3.274 | / |
| *Note*: in each cell, logistic regression coefficients, as obtained by L1 regularization, cross-validated by 5 folds. Brain regions coded as per the augmented Schaefer atlas, 232 parcels. | | | |
